# Assessment of Reinforced Concrete Surface Breaking Crack Using Rayleigh Wave Measurement

**DOI:** 10.3390/s16030337

**Published:** 2016-03-05

**Authors:** Foo Wei Lee, Hwa Kian Chai, Kok Sing Lim

**Affiliations:** 1 Department of Civil Engineering, University of Malaya, Kuala Lumpur 50603, Malaysia; ah_foo3@hotmail.com; 2 Department of Physics, University of Malaya, Kuala Lumpur 50603, Malaysia; kslim@um.edu.my

**Keywords:** reinforced concrete, surface breaking crack, surface Rayleigh wave, velocity index, amplitude index, excitation frequency

## Abstract

An improved single sided Rayleigh wave (R-wave) measurement was suggested to characterize surface breaking crack in steel reinforced concrete structures. Numerical simulations were performed to clarify the behavior of R-waves interacting with surface breaking crack with different depths and degrees of inclinations. Through analysis of simulation results, correlations between R-wave parameters of interest and crack characteristics (depth and degree of inclination) were obtained, which were then validated by experimental measurement of concrete specimens instigated with vertical and inclined artificial cracks of different depths. Wave parameters including velocity and amplitude attenuation for each case were studied. The correlations allowed us to estimate the depth and inclination of cracks measured experimentally with acceptable discrepancies, particularly for cracks which are relatively shallow and when the crack depth is smaller than the wavelength.

## 1. Introduction

Civil structures are susceptible to various kinds of defects such as cracking, spalling, creeping, honeycombing, voids and delamination of cover. Cracks are normally formed due to one or a combination of factors such as drying shrinkage, thermal contraction, restraint (external or internal) to shortening, subgrade settlement, and applied loads. Severe cracking often affects serviceability and the integrity of a structure.

Numerous studies have been conducted to assess surface breaking cracks in concrete. Sham *et al.* [1] proposed a contactless short-duration pulsed thermography Flash Thermography (FT) method for surface crack detection. It was concluded that the FT is able to detect surface cracks with widths between 0.5 mm and 1 mm. However, for smaller crack (0.1–0.5 mm) detection, addition of adding water was required. Matsuyama *et al.* [2] developed a Stack Imaging of spectral amplitudes Based on Impact-Echo (SIBIE) method to identify the presence of voids in delaminated areas and evaluate the depth of surface cracks. An extended Surface Wave Transmission (SWT) method to characterize the depths of surface-breaking cracks in concrete bridge was previously carried out by Kee and Gucunski [3]. The comparison between 3D finite element simulation and actual reinforced concrete bridge decks’ measurements was reported with an average error of 10%–15%. In addition, Yin *et al.* [4] studied a noncontact vision-based sensing method with which cracks in a full-scale reinforced concrete slab could be detected through image analysis.

The unique features of Rayleigh waves (R-waves), for example, their low attenuation and higher energy of possession than bulk waves, facilitate detection at long propagation distances. These features make R-waves a promising tool for non-destructive evaluation of concrete. The R-wave depth of propagation depends on its wavelength and it exhibits strong dispersion behavior, e.g., velocity varies with frequency of wave. Thus, for a medium with a velocity varying with depth, the R-wave velocity depends on the frequency and is known as dispersion. It is believed that the dispersion and diffraction characteristics of R-waves can be utilized to provide useful information on the propagation medium, for example, the existence of a defect [5].

With regards to applications of R-wave in non-destructive testing of concrete, Kim and Kwak [6] proposed a wavelet-component analysis technique for the measured waveforms. Its accuracy was comparable to other conventional signal processing methods, besides offering improved reliability due to successful elimination of various noises and reflection waves. Willcocks *et al.* [7] extended the existing half space theory to analyze layered structures of finite depth. It was indicated that the new proposed Spectral Analysis of Surface Wave (SASW) tool was applied to estimate the physical properties of concrete and hybrid structures of unknown layer configurations and the detection of damage in structures of known physical dimensions. On the other hand, Chai *et al.* [8] studied the feasibility of impact-generated R-waves to measure deep surface-opening cracks in concrete structures with varying vertical crack depths. The authors established correlations between the amplitude factors and crack depth-to-wavelength ratio. Subsequently, the accuracies were compared with the results of Primary wave (P-wave) time of flight method. In addition, Lee *et al.* [9] explored the possibility of a new method to determine and extract R-wave component from transient elastic waves based on an algorithm that employs matched filtering of center of energy (MFCE). The authors reported that experimental results are in good agreement with the numerical findings, confirming the feasibility of the proposed method in crack depth estimation. Alver and Ohtsu [10] examined the possibility of ultrasonic methods for subsurface damage detection in concrete specimens with varying depths. Both the numerical and experimental analyses showed R-wave and P-wave velocities were not responsive to the subsurface damage. Thus, the rendering establishment of correlations between wave attenuation rate and crack depth is still uncertain. A complementary stack imaging technique was applied for subsurface crack depth identification using ultrasonic echo. In addition, the suitability of R-waves for use in tomographic reconstruction of concrete interior was also previously studied [11]. It was reported that the single-sided measurement enabled defect visualization inside concrete, where the sensitivity relied on the penetration depth of R-waves.

The available concrete crack assessment methods were found to have their own limitations. For example, some methods only focus on detection in a qualitative manner, while others are confined to evaluating shallow cracks in plain concrete at laboratory scale, which are not sufficiently cost-effective to be adopted on a mass scale. Various issues have arisen in rationalizing the elastic wave methods, in particular to the R-waves based methods for assessment of cracks in concrete structures. In this study, the aim is to improve the R-waves based method through quantitative examination of the behavior of waves propagating concrete containing surface breaking cracks. To investigate the effect of concrete inhomogeneity on R-waves’ propagation, steel bars were included to simulate the actual reinforcement arrangement of a concrete. Key parameters of R-waves that are sensitive against changes in crack depth and degree of inclination were identified and analysed. Through regression analyses, correlations between the R-waves’ parameters and properties of cracks were obtained and validated with experimental measurements. The correlations could facilitate *in situ* measurements and characterization of surface breaking cracks.

## 2. Numerical Simulations

### 2.1. Model Description

**Figure 1:**
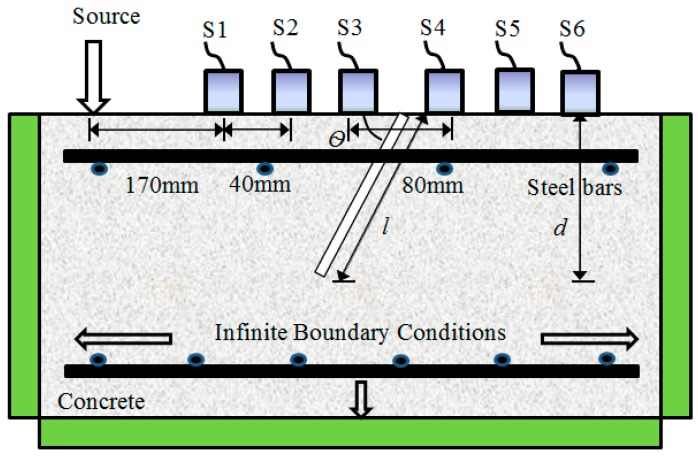
Schematic sketch of simulation model with inhomogeneity introduced by an inclined surface breaking crack.

Numerical simulations were carried out with a commercial software Wave2000 [12] that provides solutions to the two dimensional (2D) elastic wave propagation problems based on the method of finite difference. The fundamental equation governing the 2D propagation of stress waves is as follows:

(1) $$\rho \frac{\partial^{2}w}{\partial^{2}t}=\left[\mu +\eta \frac{\partial }{\partial t}\right]\nabla^{2}w+\left[\lambda +\mu +\emptyset \frac{\partial }{\partial t}+\frac{\eta }{3}\frac{\partial }{\partial t}\right]\nabla \left(\nabla •w\right)$$

where ρ is material density, λ is the first Lame constant, *μ* is the second Lame constant, η is shear viscosity, ϕ is bulk viscosity, ∇ is the gradient of operator, ∇• is the divergence operator, ∂ is the partial differential operator, *t* is the time and *w* is a two dimensional column vector whose components are the *x* and *y* components of displacement of the medium at location (*x, y*), that is:

(2) $$w={\left[wx\left(x,y,t\right)\;wy\left(x,y,t\right)\right]}^{\prime }$$

where ’ denotes matrix transpose. The time function was Sine Gaussian pulse and the specific expression used for waveform is:

(3) $$p\left(t\right)=Ae^{\left[-{\left(t-\frac{Duration}{2}\right)}^{2}/a^{2}\right)]}\sin\left(2\pi ft\right),\;t>0,\;t\ll duration$$

where *p*(*t*) = 0 for *t* ≪ 0 and *t* > *Duration*, *A* is the amplitude, the time constant *a* is inversely proportional to the bandwidth (decreasing the time constant *a* increases the bandwidth), *f* is the nominal center frequency of the waveform, and *Duration* is the time interval for which the signal is defined. A reinforced concrete model of 500 mm (width) × 300 mm (depth) in size was modelled in the simulation, as illustrated in Figure 1. The material and acoustic properties used in the model are tabulated in Table 1. An assumption has been made where the materials used are considered as elastic as all properties of concrete and rebars are uniform. Since the numerical model is a 2D model and the wave spreading is not taken totally into account, for example, geometrical spreading. To account for this, additional attenuation factors, namely shear viscosity (η) and bulk viscosity (ϕ), are considered.

Infinite boundary conditions were configured to prevent reflection of the wave from reaching the edges of the model. This is to avoid confusion during results’ analysis, since the reflected components could converge with the incoming ones, causing the actual change in waveforms. In the simulation, the input of the wave was configured as pin-point excitation on the top surface, of which the dominant frequency was varied to enable investigation of multiple cases. The simulations were implemented with one excitation that yielded a sine cycle wave propagating from one side to the other side of the model. The effect of crack on wave propagation, attenuation and pulse velocity was studied. The simulated waveform recording frequency was about 5 × 10^6^ samples per second. Three sensors were fixed on one side of the crack and another three were placed on the other side. The distance between sensors, *d*, was kept at 40 mm. The variations being investigated include angle of inclination, vertical depth, frequency of excitation and approximate R-wave wavelength, as given in Table 2. A similar procedure for the simulation can be found in [13].

### 2.2. Waveform Results

**Figure 2:**
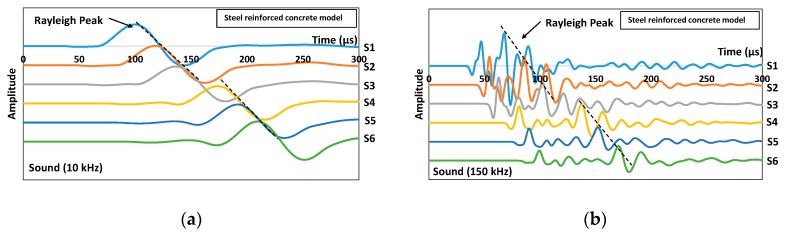

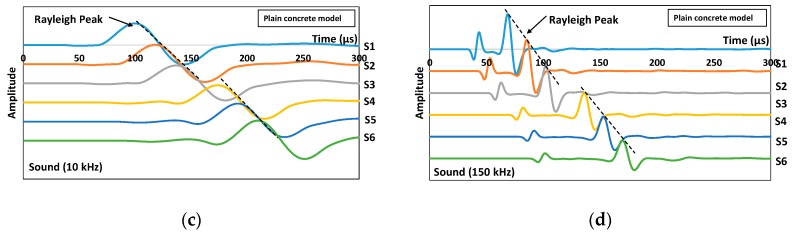
Simulated waveforms collected for steel reinforced concrete model using (**a**) 10 kHz and (**b**) 150 kHz excitations as well as the plain concrete model using (**c**) 10 kHz and (**d**) 150 kHz excitations.

The Rayleigh wave is generally detectable since it corresponds to a strong peak following the first arrival of the P-wave which is of certainly lower amplitude. In addition, R-wave velocity was also computed, which adopts the R-wave arrival time difference between the first burst peaks detected from two sensors. Figure 2 shows waveform data obtained from simulating models without crack: one composed of concrete only while the other with inclusion of steel reinforcements. The excitation frequencies used were 10 and 150 kHz. At 10 kHz excitations, the two sets of waveforms were almost identical to each other in terms of propagating speed and amplitude (Figure 2a,c), On the other hand, at 150 kHz excitations, R-waves were found to be propagating slightly faster in the steel reinforced concrete sound model, although the amplitude did not seem to differ much from those propagating the plain concrete model (Figure 2b,d). The differences between arrival times of R-waves were found to be approximately 7% at most between the two sets of waveforms with different excitation frequencies. Negligible influence by the presence of steel reinforcements on the propagation behavior of R-waves is reckoned, due to their small coverage relative to the total area by concrete in the model. Both the longitudinal and transverse reinforcements took up just 5% area of the 300 mm × 500 mm model, rendering insignificant relative displacement between steel and concrete which can be neglected. In addition, the highest frequency of the excited wave is 150 kHz, corresponding to a wavelength of 16 mm (twice the rebar diameter of 8 mm), and causing to the propagation to be insensitive to the reinforcement [14]. It is also to be noted that, in this study, the R-wave parameters, namely velocity and amplitude acquired from simulation models with cracks, would be normalized with the ones from the sound model of identical reinforcement arrangements to eliminate any possible influence on waveforms by the presence of reinforcements.

**Figure 3:**
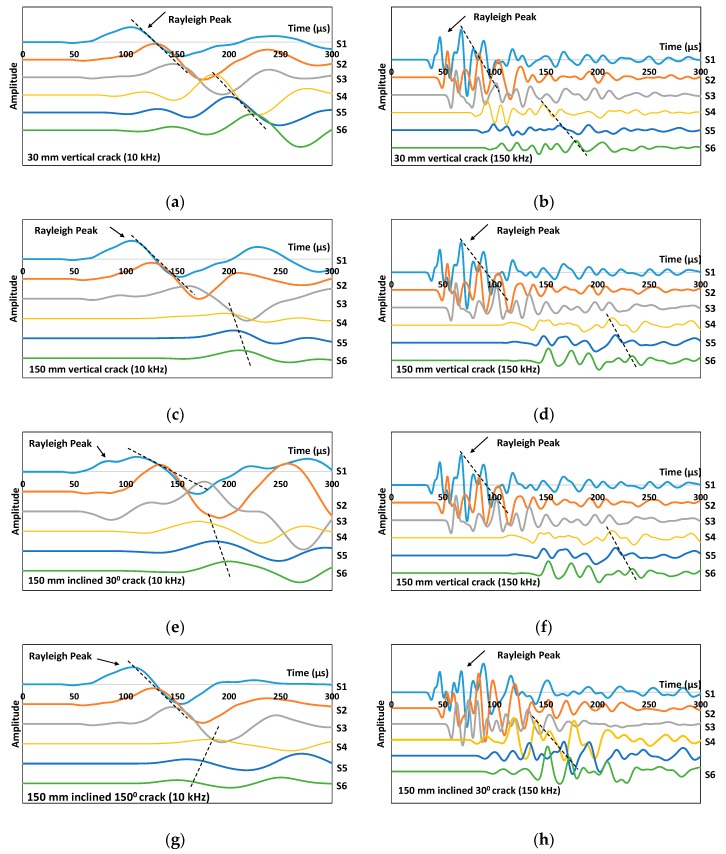
Simulated waveforms collected for 30 mm vertical (90°) crack model using (**a**) 10 kHz and (**b**) 150 kHz excitations; for 150 mm vertical (90°) crack model using (**c**) 10 kHz and (**d**) 150 kHz excitations; for 150 mm inclined 30° crack model using (**e**) 10 kHz and (**f**) 150 kHz excitations; for 150 mm inclined 150° crack model using (**g**) 10 kHz and (**h**) 150 kHz excitations.

Examples of simulated waveform results of the cracked concrete model acquired from excitation frequencies of 10 kHz and 150 kHz are given in Figure 3. The strongest cycle belongs to the Rayleigh mode, which follows the weak longitudinal arrivals that were observed, especially for higher excitation frequencies. From the figure, distorted waveforms recorded by sensors after the crack (S4, S5 and S6) indicated a larger decrease in amplitude and are hardly visible (waveforms were magnified by a factor of 5 in Figure 3f,g and a factor of 10 in Figure 3j) compared to the ones from the sensors of the homogeneous concrete model or even to those sensors before the crack (S1, S2 and S3), which are quite clear. This shows that a very small part of the energy is transmitted through the crack. The depth of the crack has greatly influenced the amplitude recorded by sensors after the crack and their relationship is found to be inversely proportional. Besides, the arrival of R-waves has obviously been delayed, especially in the deeper crack cases (150 mm) due to the longer pathway that has to be taken to the corresponding sensors. The crack acted as a void which does not allow the waves to pass through. Therefore, a longer pathway and lower velocity are expected. It is worthy to note that the orientations of the surface breaking crack can be identified from the simple arrangement of the waveforms recorded by all the sensors. For example, the waveforms obtained from sensors before the crack show a consistent delay of R-wave peak for the 90° vertical and 150° inclined crack cases, while in the case of 30° inclination cracks, distorted waveforms were recorded from sensors located before the crack as can be seen in Figure 3g,h due to the convergence between reflected body waves from the crack and the coming Rayleigh wave. In addition, one can notice that the arrival of R-wave peaks for sensors located after the crack behaves in an inverse manner for the 150° inclination crack (see Figure 3i,j). From the simulated wave motion, the generated waves were seen to travel along the crack face down to the crack tip, before being projected upward to reach the concrete top face on the other side of the model to form R-waves. The projection was complicated as observed and it would be highly possible for the wavefront energy to reach sensors S5 and S6 first depending on the crack inclination degree and depth. Under such circumstances, R-waves would propagate in two directions on the concrete top face and, sensor S4 would record its arrival later on. Hence, in the results’ analysis that will be discussed in Section 2.3, the sum of R-wave velocity from all the sensors was taken into account in order to cater for the uncertainty in the sensor acquisition sequence. It is understood from the findings that the behavior of the wave following interaction with the crack has significant angle dependences and the effect of this angular dependence is clearly defined in the R-wave arrival times. Based on this measurement configuration, the change of R-wave propagation trend could be used to identify the inclined direction of the crack. It is clear that the behavior of the wave following interaction with the defect has a significant angle dependence.

**Figure 4:**
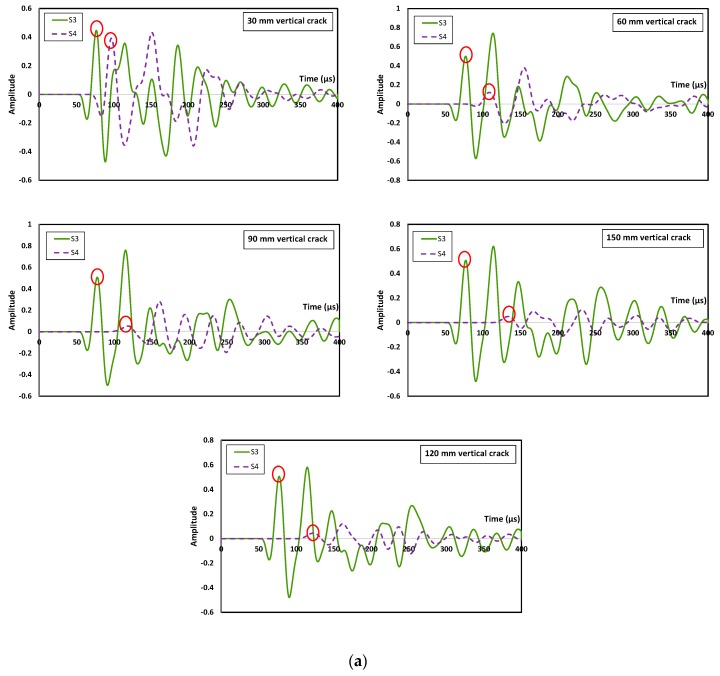

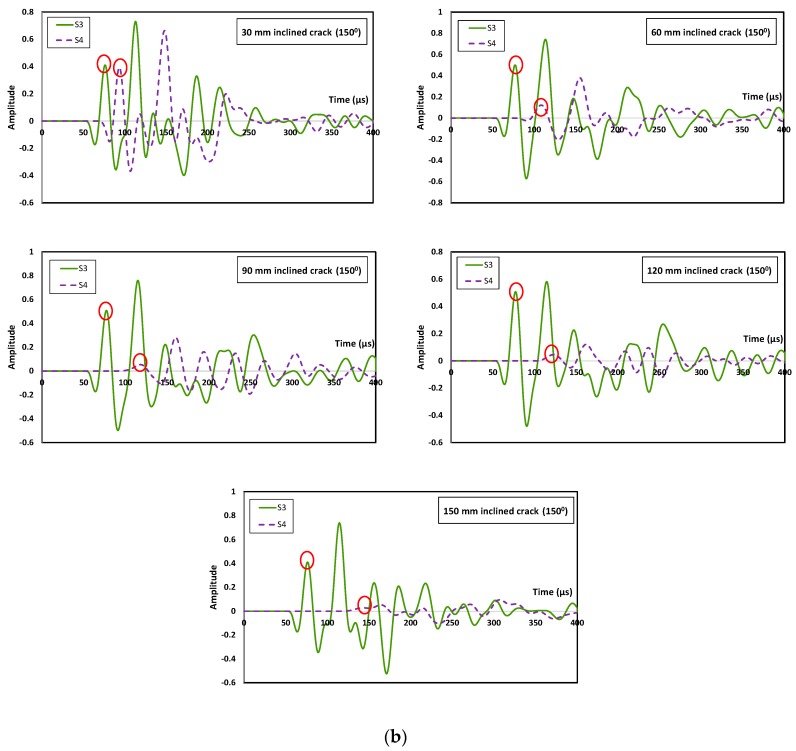
Waveforms collected at the third sensor (S3) and fourth sensor (S4) (**a**) vertical (90°) surface breaking crack and (**b**) inclined (150°) surface breaking crack for 30 kHz excitations.

Waveform data recorded from sensors S3 (left hand size of crack) and S4 (right hand side of crack) at excitation frequency of 30 kHz is depicted in Figure 4, for the vertical crack (90°) and the one inclining at 150°. For the vertical crack, waveforms obtained from the sensor on the left hand side of the crack did not exhibit much difference in terms of amplitude and R-wave arrival time. Nevertheless, for waveforms obtained by sensors at the right hand side of the crack, delay of arrival time of Rayleigh peaks as well as the decline in amplitude were noticed. The delay and decline became significant as the depth of the crack increased. From the results, it is evident that the crack depth has influence on the change in wave propagation behavior. The variations of amplitude for the shallowest crack depth of 30 mm and deepest crack depth of 150 mm were reported as 10.45%, 77.61%, 16.73% and 85.95%, respectively, for 90° and 150° cases. This is explainable since the elastic wave with a propagation frequency of 30 kHz is able to pass directly underneath the crack depth of 30 mm with minor scattering and attenuation due to its wavelength (78 mm) greater than the crack depth. Similar results are found in [15] as low frequencies pass underneath the crack, while higher frequencies which are transmitted are more likely to have travelled along the crack faces. Additionally, the corresponding percentage of delay in arrival time of R-wave peaks was calculated as 10.43%, 23.94%, 12.64% and 29.62%, respectively, for the same cases. Similar results were reported from a previous study, confirming the influence by crack depth to the velocity and amplitude of the R-waves [16].

### 2.3. Correlations of Waveform Parameters with Crack

**Figure 5:**
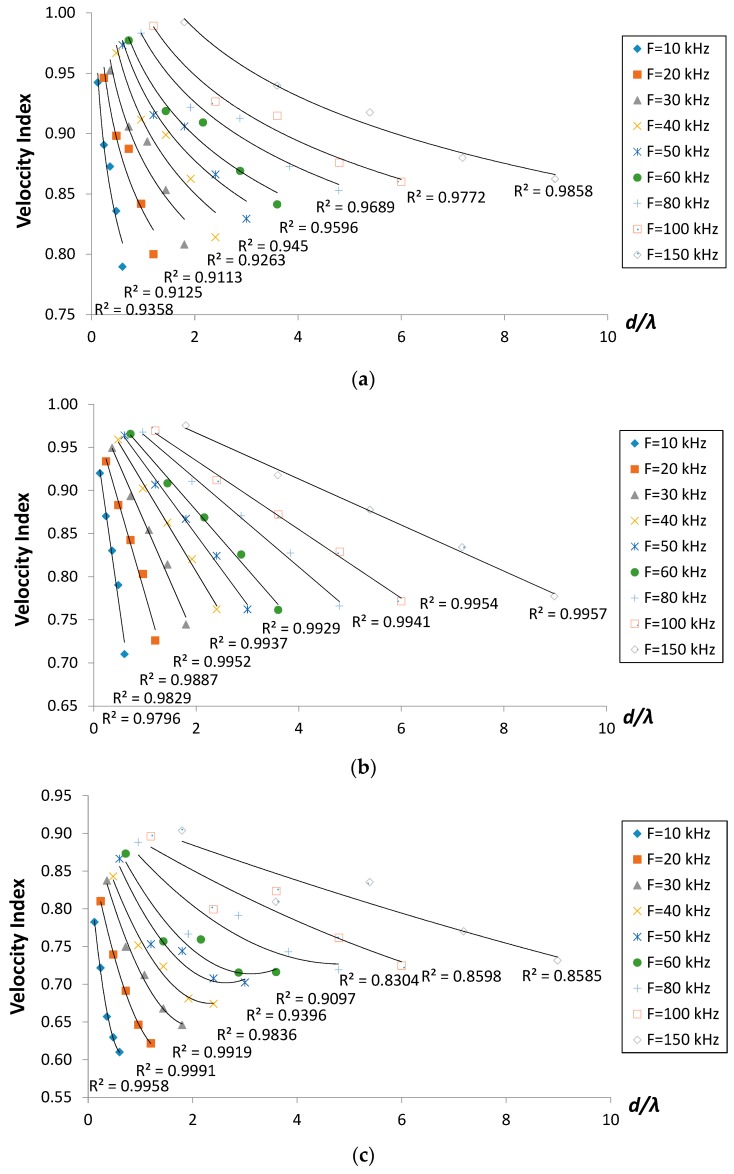
Velocity index *versus* d/λ for crack cases (**a**) 30° inclined; (**b**) vertical (90°) and (**c**) 150° inclined.

In previous researches, the crack depth is essentially divided by the major wavelength of R-waves in order to offer a parameter that gives flexibility and broader coverage in comparison and assessment [8,17,18,19,20,21,22]. In this paper, the said parameter results are presented in Figure 5. The velocity indices, *VI* for each propagation was computed using the following equation:

(4) $$VI\;=\frac{\sum_{j=2}^{6}V_{C,\;1\sim j}}{\sum_{j=2}^{6}V_{S,\;1\sim j}}$$

where $\sum_{j=2}^{6}V_{C,\;1\sim j}$ and $\sum_{j=2}^{6}V_{S,\;1\sim j}$ are summation of R-wave velocities from sensor S1 to the other respective sensors, for crack and the sound model, respectively. It is noticeable that a velocity index of 1.0 indicates that the propagation of R-waves is the same as in the sound model condition and has not been disturbed by the crack.

It is found that the velocity index decreased as the ratio of crack depth-to-wavelength, *d*/λ increased, in logarithmic regressions for crack cases of 30° and in linear regressions for the vertical one, respectively. A dissimilar trend is observed in which the velocity index decreased in polynomial regressions for cases of cracks inclining more than 90°. The figures also indicated that higher excitation frequencies will result in higher values of velocity indices. Similar findings were reported from previous studies, suggesting that although waves with higher frequencies experience stronger attenuation than lower ones, their propagation velocity is faster in inhomogeneous media like concrete [23,24] passing through the thickness transmission of P-wave, as well as R-waves [25]. Based on the findings, the velocity index could be considered as a useful parameter for identifying the existence of a crack and quantifying its depth. From the results, it is also suggested that even higher frequencies with lower penetration depth are still applicable for deeper crack identification.

**Figure 6:**
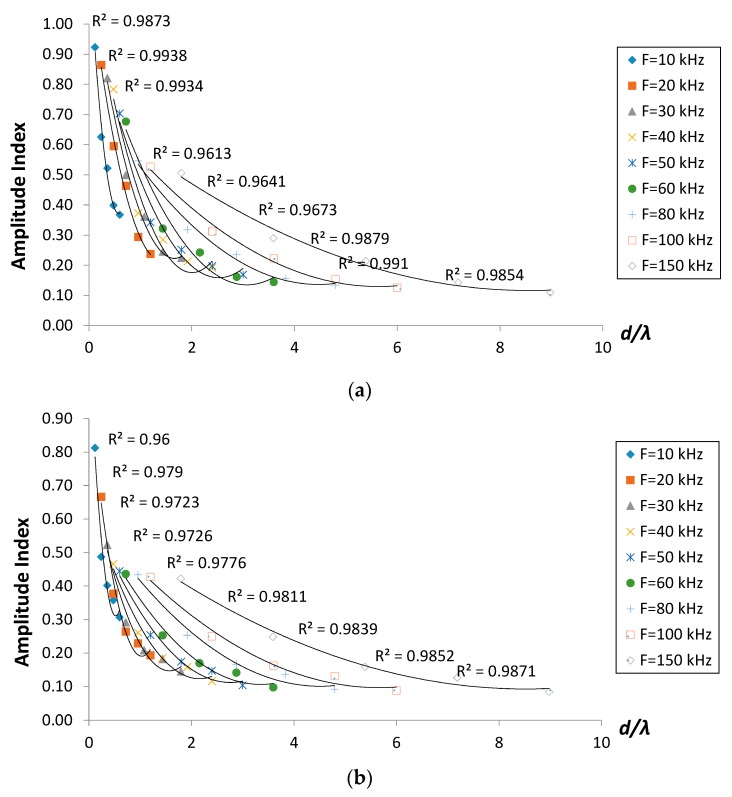

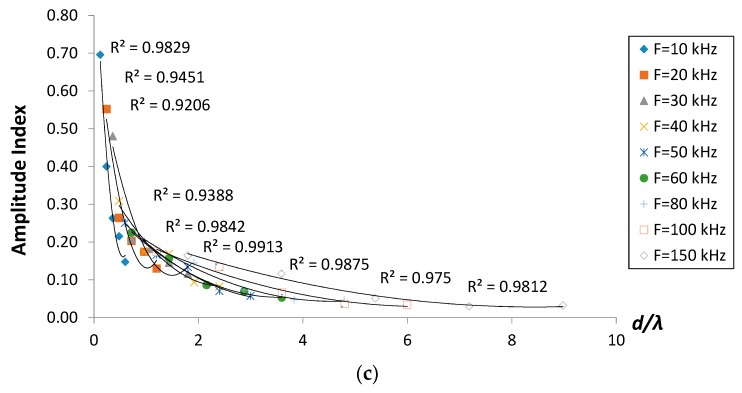
Amplitude index *versus d*/λ for crack cases (**a**) 30° inclined; (**b**) vertical (90°) and (**c**) 150° inclined.

To evaluate the effect of cracks on the amplitude of R-wave, amplitude index, *AI* (Figure 6) was calculated using the following:

(5) $$AI=\frac{\left(\sum_{i=4}^{6}A_{C,\;i}/\sum_{j=1}^{3}A_{C,\;j}\right)}{\left(\sum_{i=4}^{6}A_{S,\;i}/\sum_{j=1}^{3}A_{s,\;j}\right)}$$

where *A_c_* is R-wave amplitude in model with crack and *A_S_* is R-wave amplitude in the sound model. The amplitude index is also presented in a dimensionless form for better assessment adaptability. From the results, it is noted that the amplitude index of all crack cases decreased as the ratio of crack depth-to-wavelength, *d*/λ increased, in a polynomial regression trend. Apart from this, the amplitude index becomes lower as the frequency increases due to the tendency for higher frequency components simply to lose their energy via absorption, scattering and also attributed to distortion by the crack. Generally, the amplitude index seemed to decrease with regards to the degree of inclination from 30° to 150°. It supports the phenomenon that more energy was blocked from being detected on other side of the crack as the degree of inclination of the crack increased. The attenuation of amplitude manifested to be a more suitable parameter for crack characterization than velocity index since the discrepancy between the homogenous and cracked models was greater and more noticeable than it was for velocity. This agrees well with the previous findings [26,27].

The amplitude index also appears to lose its sensitivity towards the detection of crack depth of 150 mm. The energy of the wave is not proportional to the amplitude but to the square of the amplitude. This implies that the major energy of R-wave propagates in shallower zones. Hence, despite the fact that the penetration depth of R-waves which is considered to be one wavelength and larger than the crack, the energy passing below the crack is insignificant, and, therefore, the waveform readings for the larger cracks do not show any discrepancy [16].

### 2.4. Correlations of Waveform Parameters with Degree of Inclination

**Figure 7:**
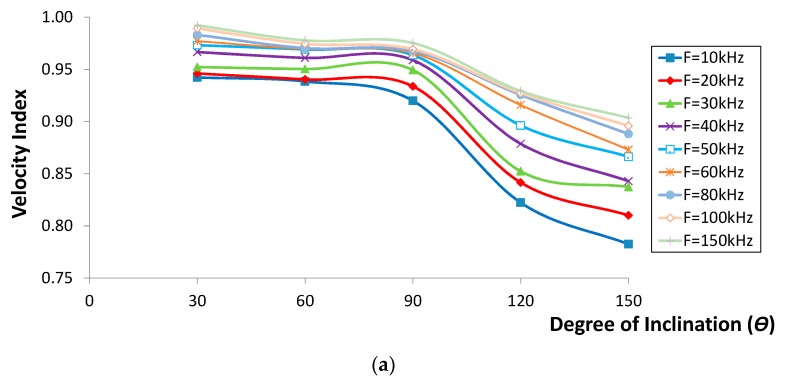

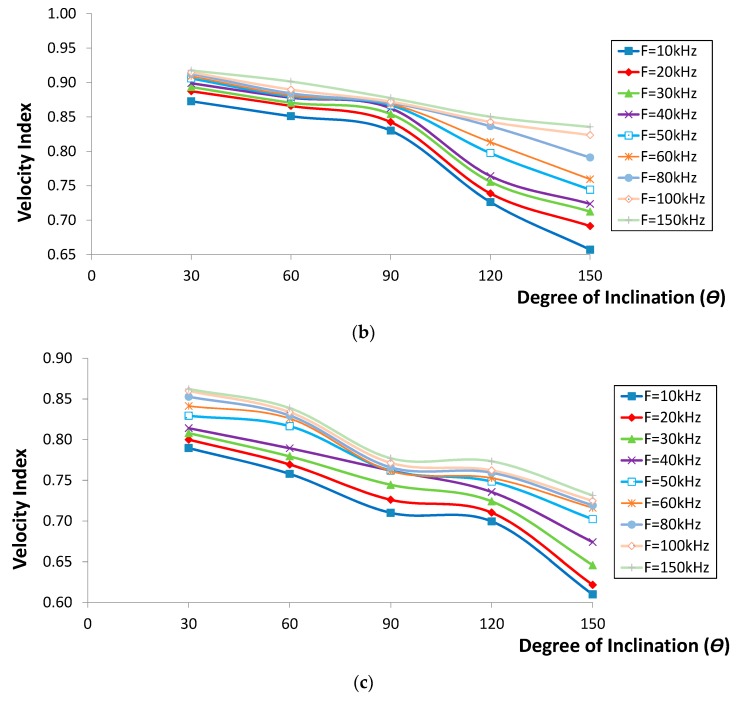
Velocity index *versus* different degree of inclination for crack depth of (**a**) 30 mm, (**b**) 90 mm and (**c**) 150 mm.

**Figure 8:**
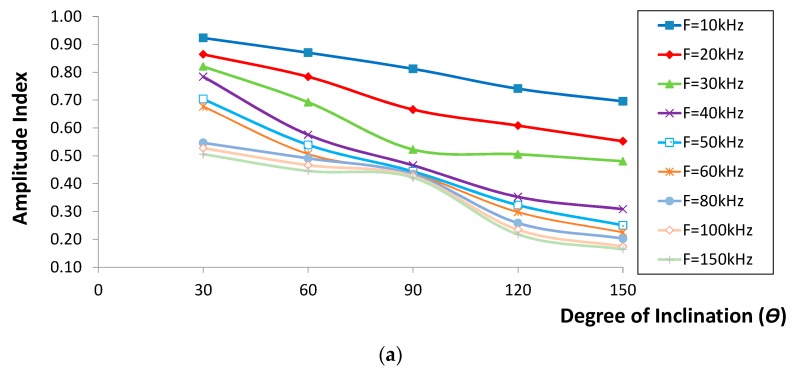

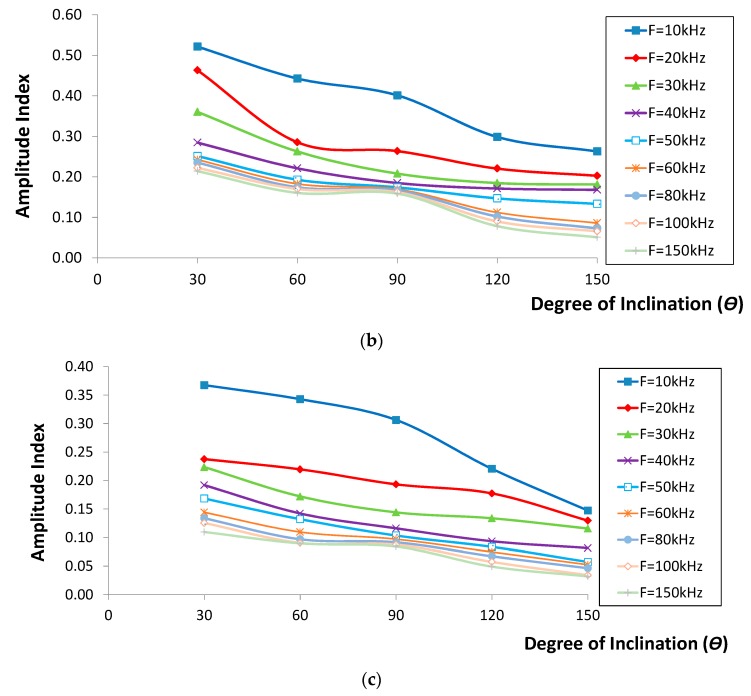
Amplitude index *versus* different degree of inclination for crack depth of (**a**) 30 mm; (**b**) 90 mm and (**c**) 150 mm.

The velocity and amplitude indices *versus* the crack inclination degree are shown in Figure 7 and Figure 8. It seems that both the velocity and amplitude indices decreased in polynomial trends as the degree of inclination increased. In addition, it can be seen that the lowest indices were obtained for the case of crack inclining at 150°, while the highest values were found from the one inclining at 30°. On the other hand, the amplitude index for the first two cases (Figure 8a,b) displayed a comparable decrease, contrary to the trend observed for other crack cases (depth of greater than 90 mm). Generally, both the velocity and amplitude indices decreased as the degree of inclination increased from 30° to 150°, proving that large energy content has been blocked due to an increase in effective vertical depth as crack inclination increased. In addition to this, it is also confirmed that the drop of velocity with the increase of crack inclination was due to the fact that R-waves, in particular those with effective penetration depth less than the crack vertical depth, took longer to propagate to the other side of the crack. It was considered that the results obtained would be of real benefit for the same measurement setup. The results may also be applied to other setup conditions with adequate adjustments, especially the scaling between the crack depth and sensor distance.

## 3. Experimental Verification

### 3.1. Specimen and Instrumentation

Four reinforced concrete specimens were prepared (300 × 300 × 500 mm). One of the specimens was the control with no defect, while the other three comprised of one artificial crack inclining at 30°, 60° and 90° (vertical crack), respectively, as measured against the horizontal plane. Additionally, 120° and 150° can be measured from the other side of the crack of 60° and 30° inclinations. The artificial crack was formed by hanging a polystyrene foam board in the concrete mould before pouring concrete. Steel bars of 10 mm in diameter were arranged in meshes of 100 × 100 mm and 170 × 150 mm at both tension and compression zones, respectively. The reinforcement meshes were placed at a depth of 50 mm from the bottom and top surfaces before casting. The concrete mixture was prepared using ordinary Portland cement, a maximum aggregate size of 20 mm, and water to cement ratio of 0.53. After casting, the specimens were cured under air-dried condition. At 28 days, the concrete achieved an average compressive strength of 31.1 MPa and a Young’s modulus of 21 GPa based on testing procedures as prescribed in BS EN 12390-3: 2002 and ASTM C469-10, using cylindrical specimens prepared in accordance with ASTM C469.

**Figure 9:**
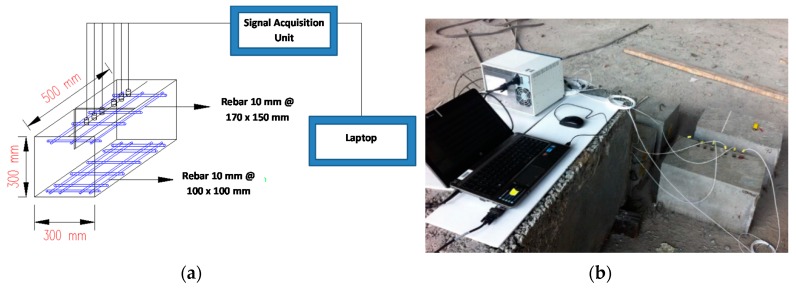
(**a**) Schematic representation of measurement setup; (**b**) photograph of laboratory measurement setup.

**Figure 10:**
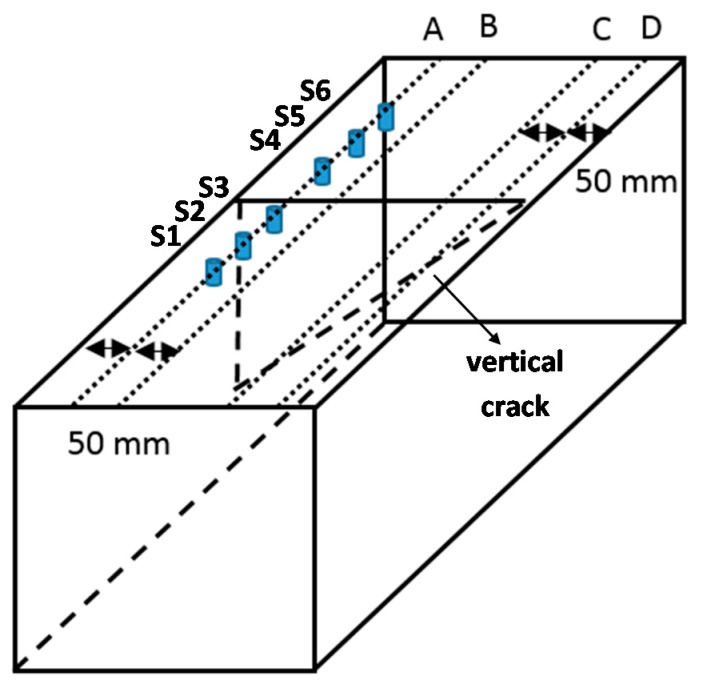
3D view of concrete specimen block and test grids over surface breaking crack.

A digital data acquisition system (NI PXIe-4492 by National Instruments Corporation) was employed in the experiment measurement. Six accelerometers (352A60 (PCB Group Inc. with a frequency range of 0.005–60 kHz were mounted on the top surface of concrete specimen using petrol gel couplant. The arrangement of sensors was identical to the configuration adopted for numerical investigations. The specimen cross sectional dimensions, density, Lamé constants and modulus of elasticity were similar to those of numerical models. Figure 9 shows the experimental set up. It was considered that any distortion of waves as recorded by accelerometers S3, S4 and S5 would be governed by the presence of the crack. In the experimental measurements, generations of waves were made by impact excitations from steel balls of different ball diameters (19 mm, 15 mm, 13 mm, 10 mm and 9 mm). The purpose was to have wave excitations of different dominant frequencies. Figure 10 shows the measurement arrays and are labeled A, B, C and D. The crack has varying depths depending on measurement location.

### 3.2. Results Discussions

**Figure 11:**
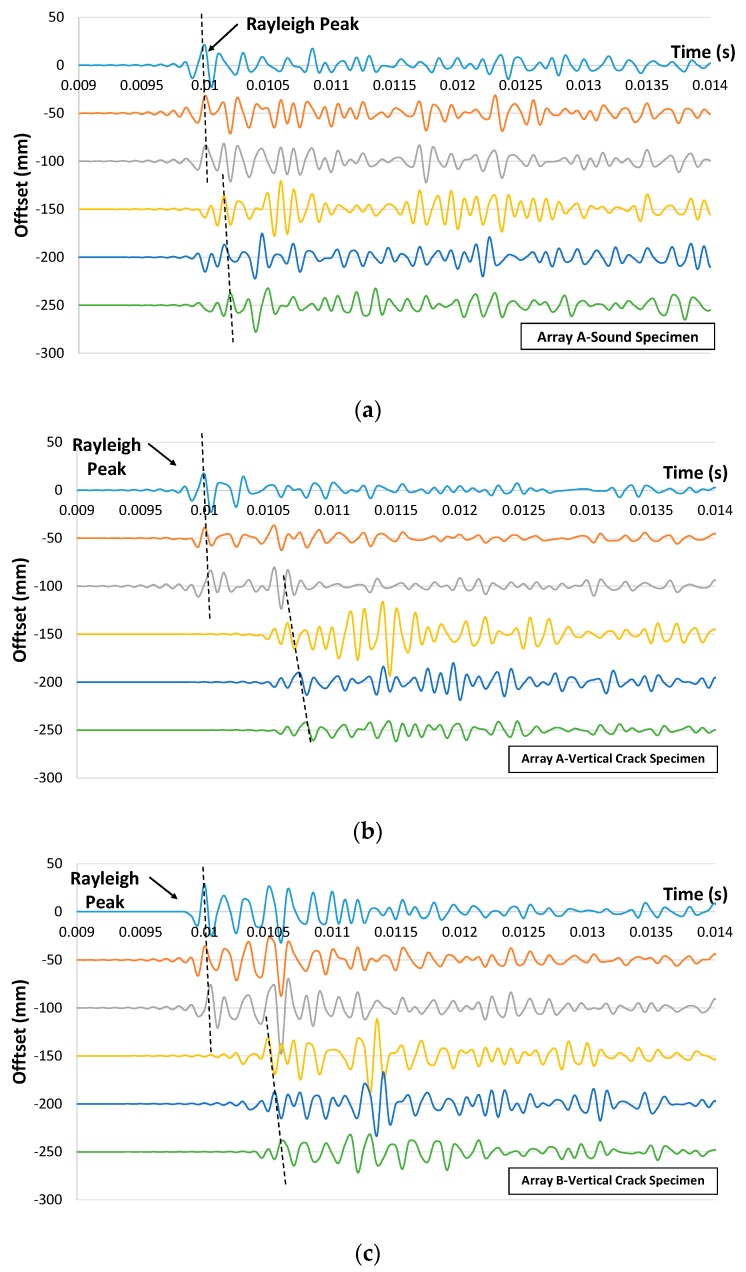

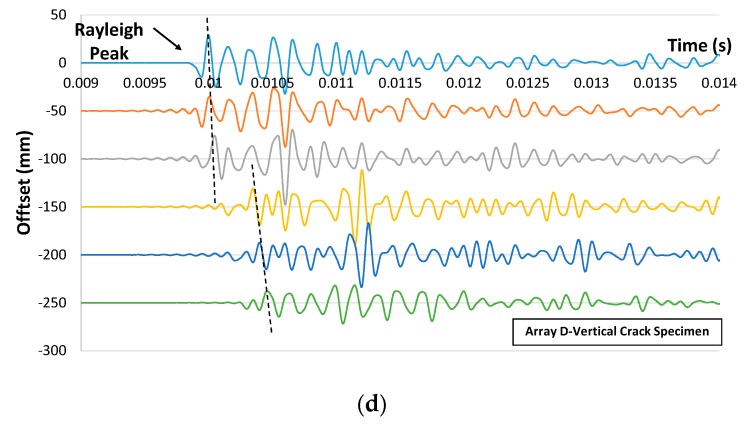
Time domain traces collected on array A for (**a**) control specimen and (**b**) specimen with vertical crack and (**c**) on array B for latter concrete specimen and (**d**) on array D for the specimen with the crack.

Steel balls with diameters of 19 mm, 15 mm, 13 mm, 10 mm and 9 mm were used as impact sources in the experimental measurements. The forcing function associated with an impact event of these balls exhibits consistent and broad spectral content with dominant frequencies of 11.1 kHz, 13.4 kHz, 15.3 kHz, 18.2 kHz and 19.5 kHz, respectively. For the control and vertical surface breaking crack (90°) concrete block specimens, excitations from both sides were considered for each array. The results were then averaged. However, only one side excitation was taken for each array for the inclined surface breaking concrete block specimen (30°, 60°, 120° and 150°) due to the intrinsic geometrical limitation. An example of the time domain traces measured on array A on the upper face of the control specimen is shown in Figure 11a. In addition, time domain traces measured at the same array location for the specimen with vertical crack (crack depth of 125 mm), and on array B (crack depth of 100 mm) as well as on array D (crack depth of 25 mm) are also depicted in Figure 11b–d, respectively. From the figures, one can notice that there is a delay in the arrival of Rayleigh peaks for all cases. Apart from that, the delay of Rayleigh peaks between S3 and S4 becomes longer when the depth of surface breaking crack is increased. From the point of view of amplitude, a significant reduction of amplitude is noticeable when comparing the amplitude recorded from sensors after the crack to those recorded from sensors before the crack. Nevertheless, due to intrinsic attenuation, reductions of amplitude are also reported for sound concrete cases. It is noted that the elastic wave might have traveled down other shorter paths in concrete medium and been diffracted by the crack tip, rather than traveling in a “straight and direct” direction underneath the crack along the measurement array. This affects mostly the first arrival of P-wave and causing it to be insignificant as compared to that coming from other travel paths and possibly produced more erroneous estimations. Similar findings were also reported in a previous study [8].

**Figure 12:**
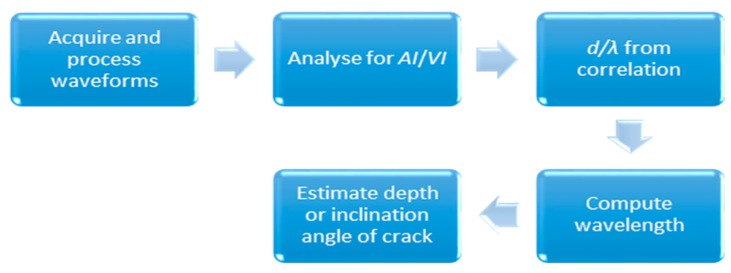
Procedure for crack depth and degree of inclination estimation.

**Figure 13:**
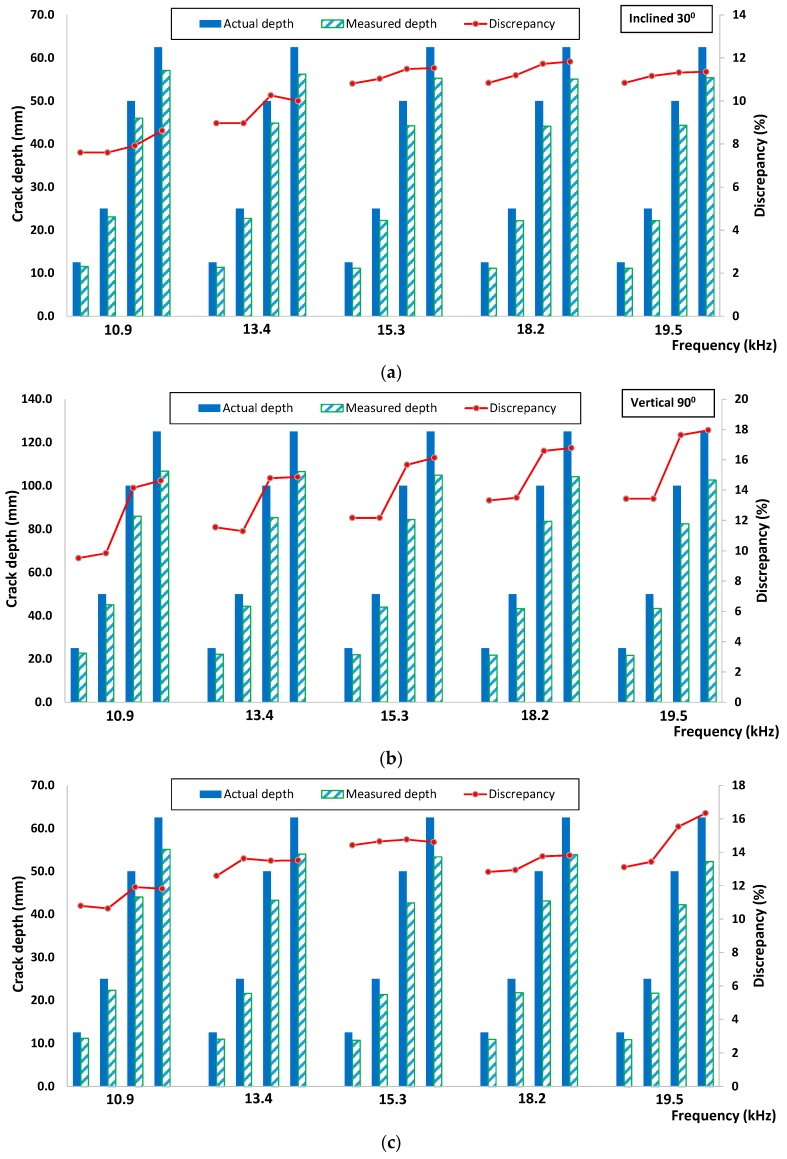
Comparison between actual and experiment measured crack depth based on velocity index for (**a**) inclined 30°, (**b**) vertical 90° and (**c**) inclined 150°.

**Figure 14:**
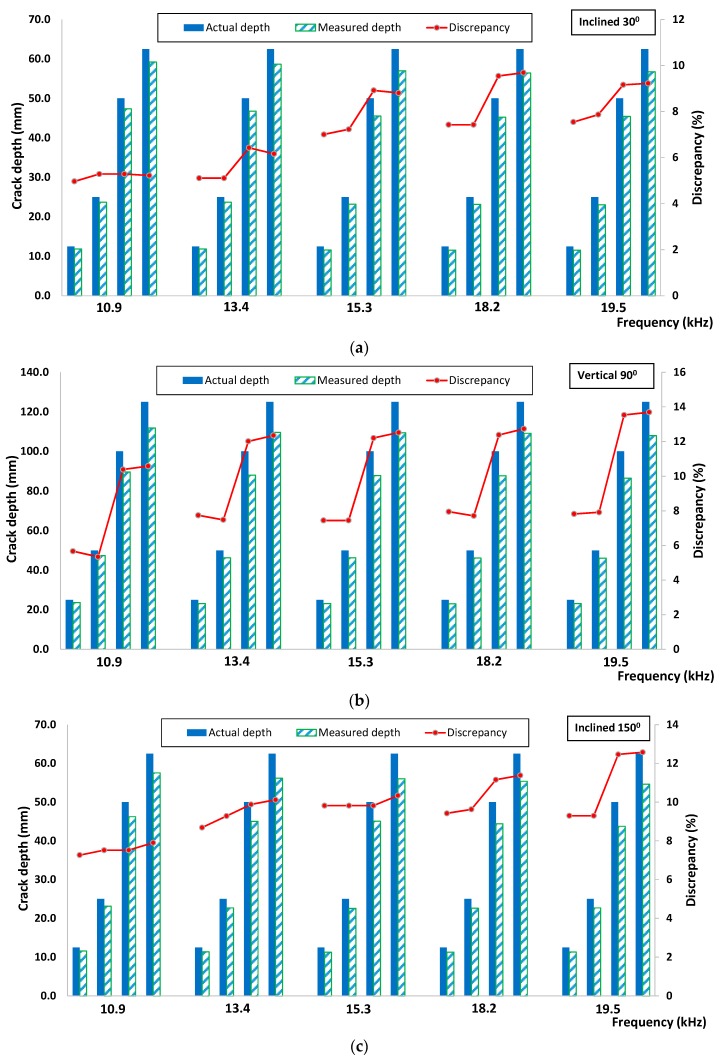
Comparison between actual and experiment measured crack depth based on amplitude index for (**a**) inclined 30°; (**b**) vertical 90°; (**c**) inclined 150°.

Figure 12 illustrated the general procedure involved in the crack depth estimation. Amplitude and velocity indices were calculated by using Equations (4) and (5) with the same signal processing and data analysis procedures as mentioned in the previous section. Since the wavelength can be known and obtained from the excitation frequency used, the estimated depth or degree of inclination can be numerically derived from *AI* and *VI* correlations obtained from simulation result analysis. The actual and experimental measured crack depths as well as their discrepancies are depicted in Figure 13 and Figure 14, based on an evaluation using velocity and amplitude indices. In general, the amplitude index provided a more accurate estimation, with discrepancies in all measurement cases being lower than those obtained from an evaluation using the velocity index. In addition, the largest discrepancy was found from measuring the specimen with vertical cracks especially for the two deeper depths (d = 100 mm and 125 mm). It is noticeable that for all cases when the propagation wavelength is smaller than the crack depths, the corresponding discrepancy values between the actual and estimated crack are lower. Apart from that, it shows that the depth of the crack was underestimated if compared to the actual ones. The propagation of elastic waves in concrete medium could be more complicated compared to the 2D simulation; the elastic wave may have been propagated down other shorter pathways in concrete medium and diffracted by the crack tip, rather than traveling in a “straight and direct” direction from below the crack. However, the discrepancy between estimated and actual depths/degree of inclination was within ±18%, suggesting that the proposed method could be useful for crack depth and degree of inclination estimation.

**Figure 15:**
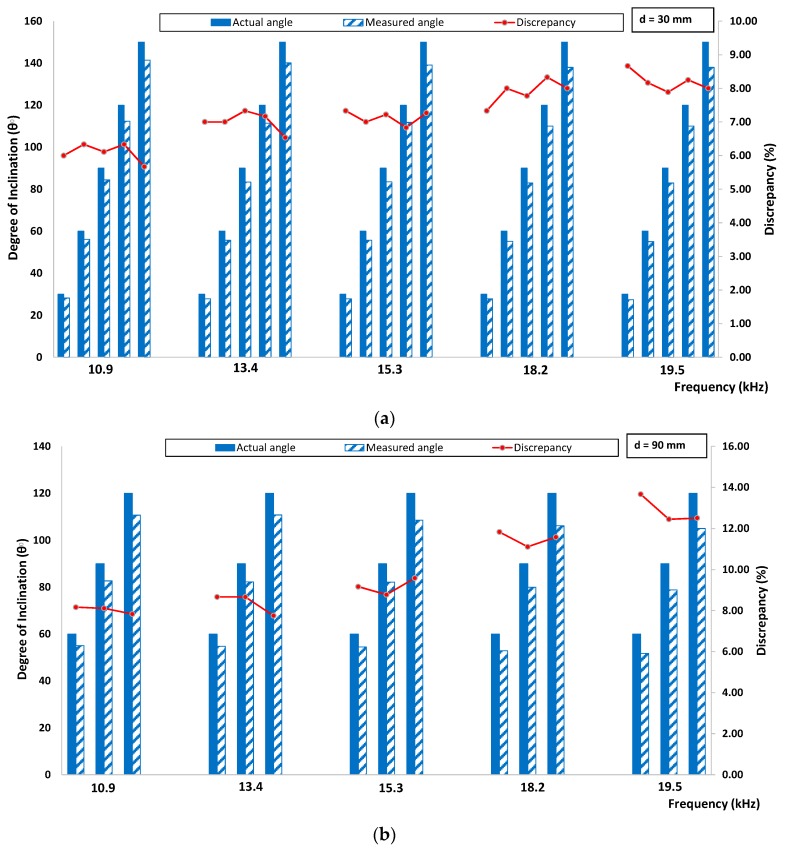
Comparison between actual and experimentally measured degree of inclination based on velocity index for (**a**) 30 mm crack depth and (**b**) 90 mm crack depth.

**Figure 16:**
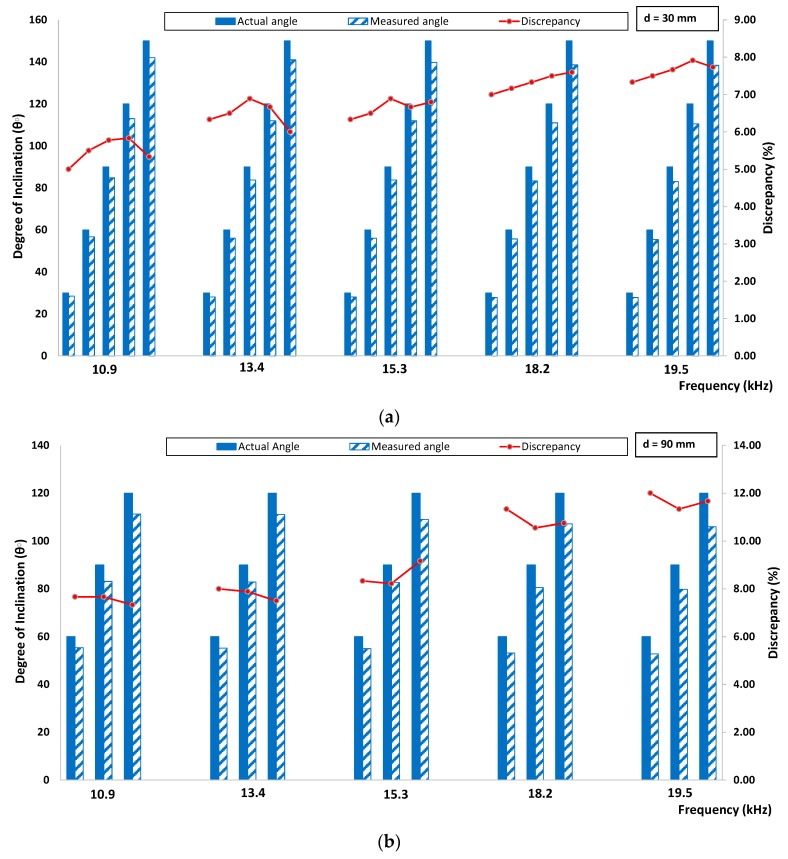
Comparison between actual and experimentally measured degree of inclination based on amplitude index for (**a**) 30 mm crack depth and (**b**) 90 mm crack depth.

Figure 15 and Figure 16 show the discrepancy between actual and experimentally measured degree of inclination for velocity and amplitude indices, respectively. It can be seen from the figure that the determination of the degree of inclination of surface breaking crack was greatly influenced by the crack depth. Few factors contributed to the discrepancy. First, the sample surface is relatively flat, but still has a slight local curvature; this can lead to incomplete and inconsistent contact between the surfaces of the accelerometers and the specimen. Second, even though the contact pressure on the accelerators is maintained as constant as possible, the contact condition varies in each measurement due to the inherently porous nature of the concrete surface. Third, the specimen has localized minor surface cracks or unseen voids that are possibly due to the hardening of concrete binder over time. In addition, the discrepancy can be due to the intrinsic attenuation caused by the material since the concrete is known to be an inhomogeneous material and geometrically spreads when the waves pass through the specimen. Finally, the variation of the signal shape due to the above-mentioned error sources causes some difficulty in consistent windowing of the signals.

## 4. Conclusions

The interaction of wave propagation with the surface breaking crack is studied both numerically and experimentally in this paper. The study is focused on the determination of the surface breaking crack depth and also its degree of inclination. Two parameters—namely, velocity and amplitude—were calculated and extracted from simulated time domain waveforms and the corresponding established correlations were verified through experimental measurements. The overall results exhibit a good qualitative agreement regarding the relationship between the proposed parameters and surface breaking crack depth as well as the degree of inclination with a maximum discrepancy of around 16%. From the analyses, the amplitude index seemed to be more sensitive towards the changes in crack depth and also the degree of inclination, especially when the wavelength is greater than the crack depth due to its higher penetration ability. Apart from the proposed wave parameters used for crack characterization, the feasibility of other parameters, such as the central frequency, peak frequency, coherence, the cut off frequency and phase velocity, are worth exploring. In addition, the parameters used for simulations can be better defined to overcome the discrepancies between the actual and measured values such as the intrinsic geometrical attenuation and the viscoelasticity of concrete materials as well as the three-dimensional (3D) propagation behavior of elastic waves. An interesting future work could focus on the neural network for simultaneous identification of crack depth and degree of inclination. Apart from this, a real site investigation, especially for a large concrete specimen, could look at different sizes or forms of cracking or deterioration. It is recommended that cross-checking the proposed parameters will enhance their reliability in crack characterization.

## Figures and Tables

**Table 1:** Mechanical properties of the model materials obtained from experimental measurements.

Material	First Lame Constant, λ_m_ (GPa)	Second Lame Constant, µ_*m*_ (GPa)	Density, ρ (kg/m^3^)	Poisson Ratio, *v*	P-Wave Velocity, *C_P_* (m/s)	R-Wave Velocity, *C_R_* (m/s)
Concrete	10.82	15.98	2313	0.202	4300	2311
Steel	124.82	83.59	7850	0.299	6099	3219

**Table 2:** Orientation, degree and depth of cracks.

Depth of Crack, *d* (mm)	Frequency of Wave, *F* (kHz)	Corresponding R-Wave Wavelength, λ (mm)	Degree of Inclination θ against the Horizontal Plane, (°)
30 to 150 at 30 increment	10, 20, 30, 40, 50, 60, 80, 100, 150	221, 114, 78, 59, 47, 39, 29, 24, 16	30, 60, 90, 120, 150
